# Serum glomerular albumin permeability activity: association with rapid progression to end-stage renal disease in focal segmental glomerulosclerosis

**DOI:** 10.1186/s40064-016-2077-9

**Published:** 2016-04-11

**Authors:** Sudhindra Pudur, Tarak Srivastava, Mukut Sharma, Ram Sharma, Sergey Tarima, Hongying Dai, Ellen T. McCarthy, Virginia J. Savin

**Affiliations:** Renal Hypertension Center, Hudson, FL USA; Section of Nephrology, Children’s Mercy Hospital, University of Missouri at Kansas City, 2401 Gillham Road, Kansas City, MO 64108 USA; Kansas City Veterans Hospital, Kansas City, MO USA; Division of Biostatistics, Medical College of Wisconsin, Milwaukee, WI USA; Kidney Institute, University of Kansas Medical Center, Kansas City, KS USA

**Keywords:** Permeability activity, Focal segmental glomerulosclerosis, Renal replacement therapy

## Abstract

**Background:**

Focal segmental glomerulosclerosis (FSGS) is a major cause of renal failure. Sera of some FSGS patients increase glomerular albumin permeability (P_alb_) during in vitro testing and cause proteinuria in experimental animals.

**Objectives:**

To determine whether permeability activity of FSGS serum (P_alb_ activity) is associated with rate of progression to renal replacement therapy (RRT).

**Design:**

This is an observational study based on medical and demographic information and P_alb_ activity testing.

**Setting:**

Studies were performed at Medical College of Wisconsin.

**Patients:**

Serum was submitted by patients’ nephrologists for measurement of P_alb_ activity. Each patient had had a biopsy diagnosis of FSGS, had reached ESRD and was on dialysis or had a functioning transplant.

**Measurements:**

P_alb_ activity, clinical characteristics and time between biopsy diagnosis and RRT (T-RRT) were recorded for each patient.

**Methods:**

P_alb_ activity was measured using established in vitro techniques.

**Results:**

P_alb_ and T-RRT were inversely correlated. Neither P_alb_ nor T-RRT varied with demographics or medications. Kaplan–Meier survival curves showed that patients with P_alb_ ≥ 0.5 progressed to RRT more rapidly than others.

**Limitations:**

Only patients who had reached RRT were included. Limited clinical information was available for each patient. Central verification of biopsy characteristics was not performed and detailed descriptions of renal histology were not available.

**Conclusions:**

P_alb_ activity is associated with the rate of progression to RRT in patients with FSGS. Additional observations will be needed to verify that P_alb_ activity predicts prognosis and is useful in stratifying patients for clinical decision making or treatment trials.

## Background

Focal segmental glomerulosclerosis (FSGS) is characterized by nephrotic-range proteinuria and a distinctive histopathological appearance on biopsy. FSGS accounts for a third of primary nephrotic syndrome in adults and a quarter in children. The etiology of FSGS is unknown in the majority of cases, but can be secondary to genetic abnormalities, decreased nephron number, hyperperfusion/hyperfiltration, reflux nephropathy, obesity, viral infections, drugs and malignancy (Daskalakis and Winn 2006; D’Agati et al. 2011). The lesion in FSGS is progressive and leads to a need for renal replacement therapy (RRT) in 50–75 % of patients over a 10-year period (Braun et al. 2008; Korbet 2012; Franceschini et al. 2003; Burgess 1999). In a subset of patients with FSGS, the progression to RRT is rapid and progression to end-stage renal disease occurs within 3 years. A measure by which one could identify patients who are at high risk for rapid progression to RRT would allow modification of the approach to therapy for the individual patient. In addition, a tool to identify these subjects would be valuable in designing and interpreting clinical trials on FSGS.

We have developed albumin permeability (P_alb_) testing as a functional assay to provide a sensitive measure of the integrity of the glomerular macromolecular permeability barrier. Experimental values for P_alb_ activity vary from 0, normal, to 1.0, representing maximal loss of barrier function (Savin et al. 1992). We have used this test to determine the effect of patient serum on glomerular barrier function. We have shown increased permeability activity (P_alb_ activity or P_alb_) in samples of serum or plasma from renal transplant recipients with recurrent FSGS (Cattran et al. 2003; Savin et al. 2008). P_alb_ activity is stable over time (Cattran et al. 2003; Savin et al. 1996, 2008) and is not affected by treatment with cyclosporine even when there is remission of nephrotic syndrome and is diminished by plasmapheresis (Savin et al. 1996). The injurious effect of serum as well as the occurrence of proteinuria in experimental animals after injection of serum, plasma or plasma fractions, and remission of post-transplant proteinuria after plasmapheresis or immunoadsorbtion has led us to hypothesize that a circulating factor is responsible for recurrence of FSGS in allografts (Savin et al. 1996; Sharma et al. 2002, 2004a, b; McCarthy et al. 2010; Zimmerman 1984). The aim of the current study was to determine if high P_alb_ activity in patients with FSGS is associated with rapid progression to RRT in native kidneys.

## Methods

### Patient samples

Albumin permeability (P_alb_) activity was determined using serum samples submitted to the Center for Glomerular Pathophysiology at the Medical College of Wisconsin in the course of clinical evaluation of FSGS. All research activities were approved by the Human Subjects Committee of the Medical College of Wisconsin and were in compliance with the Helsinki Declaration. Samples were frozen at the site of collection and shipped to the study site by overnight carrier and remained frozen until they were thawed for analyses. Samples submitted from January 2001 through December 2004 were included. Inclusion criteria were diagnosis of primary FSGS as indicated on renal biopsy report, progression to renal replacement therapy (RRT) and availability of data including patient demographics, laboratory data, renal biopsy diagnosis, date of renal biopsy, date of initiation of dialysis or transplant and medications prescribed at the time the serum specimen was collected. Data regarding the duration of symptoms and information about medical therapy given prior to serum sample collection were not available. Patients with incomplete data and those who had not progressed to RRT were excluded. Values from samples obtained after plasmapheresis therapy were excluded because we have shown that this therapy decreases P_alb_ activity (Savin et al. 1996). No information regarding renal function at the time of the biopsy, reason for performing the biopsy, details of renal histology, or classification of the pattern of FSGS was available. Time to renal replacement therapy (T-RRT) was defined as the duration between the date of biopsy diagnosis of FSGS to one of the following: (a) the date of initiation of dialysis, (b) the date of renal transplantation or (c) the date when GFR was <15 ml/min as estimated by MDRD formula for adults and Schwartz formula for children. Rapid progression was defined as the progression to RRT within 3 years of diagnosis. This cut-off was chosen because it is commonly used in the pediatric literature as a parameter associated with high risk for post-transplant recurrence (Artero et al. 1994).

### Albumin permeability (P_alb_) assay

P_alb_ is a continuous dimensionless variable that ranges from 0 to 1. P_alb_ testing was carried out under standard conditions (Savin et al. 1992, 1996). Briefly, glomeruli were isolated from the renal cortex of normal rats. Isolation/incubation medium was isotonic and contained bovine albumin, 5 gm/dl, as an oncotic agent. Glomeruli were incubated for 10 min at 37 °C in medium to which patient serum, 2 % vol/vol, had been added or in control medium. P_alb_ was calculated as previously described (Sharma et al. 2000). In the current study, P_alb_ activity of ≥0.5 was used to define high activity. This cutoff value is consistent with our practice for interpretation of values for individual patients and is the 95 % confidence limit for values from normal serum or sera of patients with non-glomerular disease (Savin et al. 1996). We performed additional analyses in which patients were stratified using P_alb_ activity of ≥0.4 to ≥0.7. All measurements were carried out without knowledge of the sample being tested and both positive and negative control samples were tested in each assay.

### Statistical analyses

Continuous variables were summarized as mean ± SD. Univariate correlations were evaluated using Pearson correlation analysis. Group comparisons were performed using Student’s *t* test. The Chi squared test was used to investigate changes in proportion of categorical variables. Odds ratio was used to measure the association of P_alb_ with T-RRT. Renal survival was investigated using Kaplan–Meier survival curves stratified by time compared between groups using the log-rank test. In addition, a latent class regression model was fitted to the data to detect heterogeneity of associations between P_alb_ and T-RRT. Statistical analysis was performed using SPSS 20.0 software (SPSS Inc., Chicago, IL) and SAS version 9.1(SAS Institute Inc., Cary, NC). A p-value of <0.05 was considered statistically significant.

## Results

### Patient characteristics

A total of 103 serum samples were submitted during the period studied. Of these, 87 were from patients with FSGS. The others were from patients in whom no biopsy report was available or who had other proteinuric renal diseases. Complete data was available for 80 samples; these were included in further analysis. P_alb_ activity averaged 0.58 ± 0.25 (mean ± SD, n = 80, range 0.05–0.98) with a median of 0.52. Patients’ age ranged from 1 to 63 years; 35 (44 %) were male, 53 (66 %) were Caucasian, 21 (26 %) were African-American, 5 (6 %) were Hispanic and 1 (1 %) Asian. Average T-RRT was 3.2 ± 3.3 years (n = 80, range 0–14.5 years). At the time of sample collection, 25 % were receiving angiotensin converting enzyme inhibitors (ACE-I), 11 % angiotensin receptor blockers (ARB) and 16 % statin therapy (Table 1). Sixteen percent were receiving steroids, 5 % cyclosporine. Eighteen patients had been transplanted, 6 had experienced recurrence and 4 had lost allografts. Seven of the transplanted patients were not on immunosuppression at the time of the sample; each of these patients had failing allografts or had lost prior allografts. All patients who experienced FSGS recurrence had P_alb_ activity ≥0.5.

**Table 1:** Patient characteristics

	All patients	Stratified by P_alb_		Stratified by T-RRT	
Number	80	P_alb_ < 0.5 25	P_alb_ ≥ 0.5 55	T-RRT < 3 years 45	T-RRT > 3 years 35
Age (years)	30.1 ± 17.2	29.7 ± 19.6	30.2 ± 16.2	30.3 ± 17.0	30.62 ± 16.7
Male gender, n (%)	35 (44)	10 (40)	25 (45)	20 (44)	15 (43)
Ethnicity, n (%)					
Caucasian	53 (66)	17 (68)	36 (65)	26 (47)	27 (76)
African American	21 (26)	6 (24)	15 (27)	15 (33)	6 (17)
Other	7 (9)	2 (8)	5 (9)	1 (0.4)	6 (13)
Medications, n (%)					
ACEI and/or ARB	25 (31)	7 (28)	18 (33)	15 (33)	10 (28)
Steroids	13 (16)	1 (4)	12 (21)	7 (16)	6 (17)
Cyclosporine or tacrolimus	4 (5)	1 (4)	3 (5)	7 (16)	6 (17)
Statins	13 (16)	3 (12)	10 (18)	9 (20)	4 (11)

Number of patients in each category is as indicated. Percentages are calculated according to the number in each P_alb_ or T-RRT category

There were no statistically significant differences between patients with P_alb_ < 0.5 and those with higher values nor were there differences between those with T-RRT < 3 years and those with a longer course

### Relationships between P_alb_ activity and T-RRT in FSGS

**Fig. 1:**
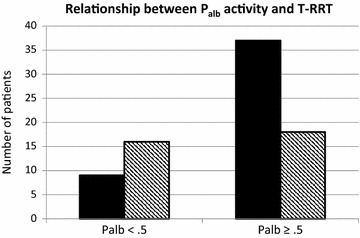
Depiction of the relationship between P_alb_ activity and T-RRT for patients with P_alb_ ≥ 0.5 and P_alb_ < 0.5 (n = 80). *Black bars* indicate patients in whom T-RRT was less than 3 years. *Hatched bars* indicate patients in whom T-RRT was 3 years or greater. P = 0.009, Chi square test, as described in the text. The odds ratio for T-RRT < 3 years in patients with P_alb_ activity ≥0.5 was 3.65 (95 % CI of 1.36–9.86)

Forty-six patients (57.5 %) required RRT within 3 years after renal biopsy. P_alb_ activity in these patients was significantly higher than in patients who had a longer course to RRT (0.63 ± 0.22 vs. 0.50 ± 0.28, p = 0.025). In patients with T-RRT > 3 months, there was a significant inverse correlation between P_alb_ activity and T-RRT described by the equation T-RRT = 7.10 − 4.96*P_alb_ (Pearson correlation coefficient r = −0.27, p = 0.016). P_alb_ activity was the only significant predictor for T-RRT. There was no effect of age, gender or ethnicity (proportional hazards model). The frequency distribution of P_alb_ activity ≥0.5 and T-RRT was calculated in order to further examine the association between P_alb_ activity and T-RRT. Results are shown in Fig. 1. Associations were as follows: Thirty-seven patients had T-RRT < 3 years and P_alb_ ≥ 0.5, 9 had T-RRT < 3 years and P_alb_ activity <0.5, 18 had T-RRT ≥ 3 years and P_alb_ activity ≥0.5 and 16 had T-RRT ≥ 3 years and P_alb_ activity <0.5. The odds ratio was calculated for T-RRT < 3 years in patients with P_alb_ activity ≥0.5 was 3.65 (95 % CI of 1.36–9.86). Chi square test showed a significant difference on T-RRT < 3 years between low and high P_alb_ activity groups (36.0 vs. 67.3 %, respectively, p = 0.009). There were no significant differences in P_alb_ between Caucasians and African-Americans (p = 0.76) or between genders (p = 0.64). P_alb_ activity did not vary significantly with medications, age, gender or ethnicity as covariates in a linear regression model.

**Fig. 2:**
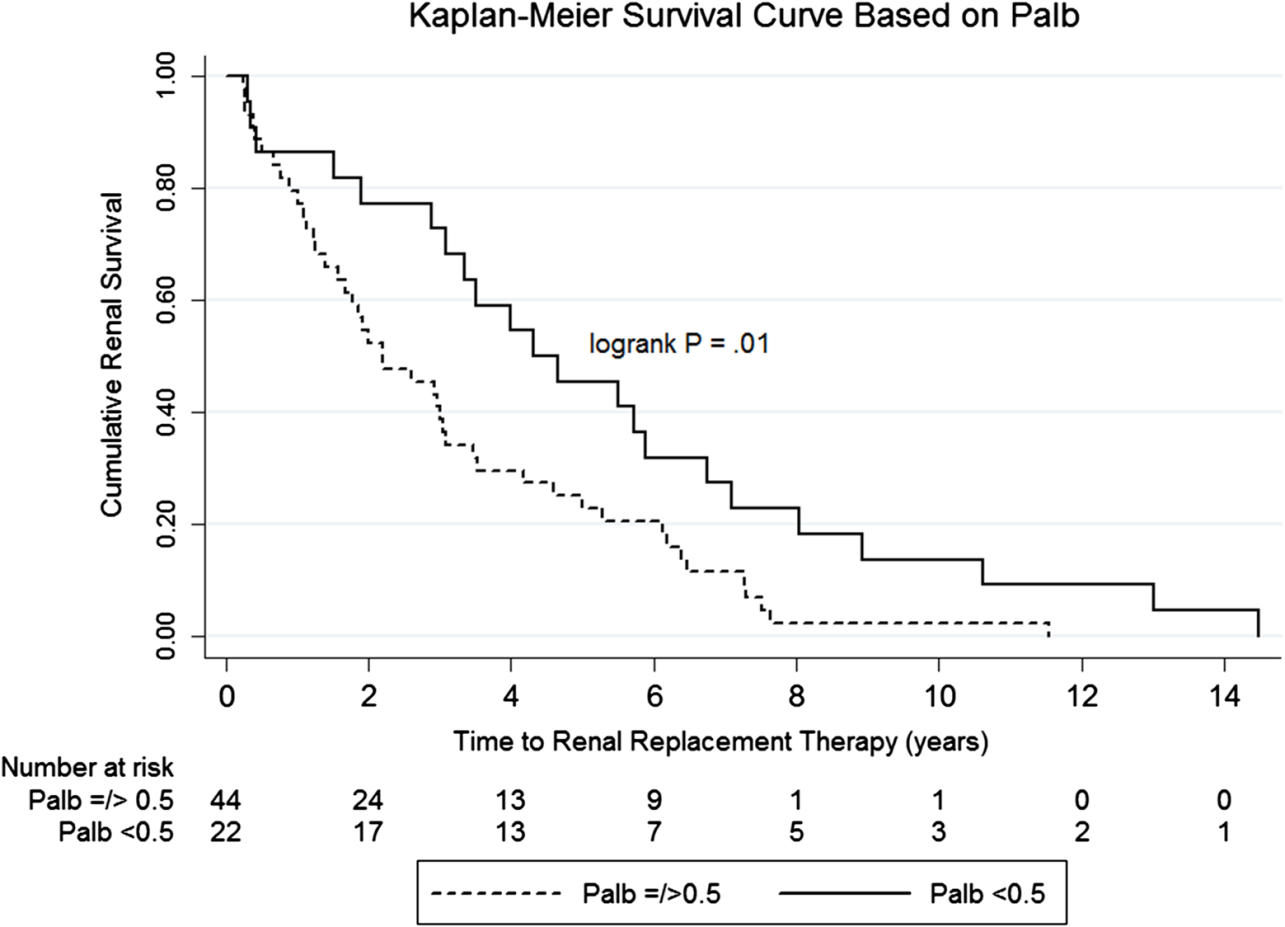
Kaplan–Meier survival curve. Patients with focal segmental glomerulosclerosis (FSGS) were stratified by albumin permeability (P_alb_) ≥0.5 (*solid line*) and P_alb_ < 0.5 (*dashed line*). Kaplan–Meier survival curve showed that patients with who had P_alb_ ≥ 0.5 progressed to RRT more rapidly. Patients with calculated T-RRT < 3 months (0.25 years) were excluded from this analysis (see text in “Results” section, “latent class regression model” for the rationale for this exclusion)

Kaplan–Meier survival curves showed that patients who had P_alb_ ≥ 0.5 progressed to renal replacement therapy more rapidly than others by log rank (Mantel–Cox) test (p = 0.009). The Kaplan–Meier curves displayed here were generated using only samples from patients (n = 66) in whom T-RRT was >3 months (Fig. 2). This decision was made on the basis of the latent class regression model described below. Note that the survival diverged after 1 year and that by 4 years, survival was more than double in the group with low P_alb_ activity versus those with high P_alb_ activity. Differences in the Kaplan–Meier survival curves were also significant for P_alb_ ≥ 0.4 (p = 0.01) and P_alb_ activity ≥0.6 (p = 0.012), but not for a P_alb_ activity cut-off ≥0.7 (data not shown). Thus, P_alb_ activity discriminated between patients with FSGS who did or did not have T-RRT within 3 years. At a cut-off for P_alb_ activity of ≥0.5, the sensitivity and specificity were 80.4 and 47.1 %, respectively. The positive and negative predictive values were 67.3 and 64.0 %, respectively.

### Latent class regression model

A latent class regression model for associations between P_alb_ and T-RRT was applied and 2 distinct patterns were identified. In 14 patients, T-RRT was ≤3 months. These patients had advanced CKD at the time of biopsy as indicated by high serum creatinine. P_alb_ activity was not significantly associated with T-RRT. In the remaining 66 subjects, in whom biopsies showing FSGS were performed when the GFR was higher, there was an inverse association between P_alb_ and T-RRT described by the fitted regression equation, T-RRT = 7.10 − 4.96*P_alb_.

## Discussion

The clinical course in adults with primary FSGS is variable, with about half of adults progressing to RRT in 10 years (Braun et al. 2008; Korbet 2012; Franceschini et al. 2003; Burgess 1999). Currently, there is no good histological or biochemical marker that predicts outcome in FSGS. Histological variants have been described and attempts have been made to use histological classification to predict response to therapy and prognosis (Schwartz et al. 1999; Thomas et al. 2006; Chun et al. 2004; Barisoni 2012). The collapsing variant shows a more rapid progression compared to other histological classes but other histological variants including tip lesion do not predict either response to treatment or prognosis (Valeri et al. 1996; Detwiler et al. 1994; Couser 2005). The treatment of primary FSGS is based on the rationale that a humoral factor or factors derived from a dysregulated immune system are responsible for development of proteinuria. At present, the majority of therapeutic interventions depends on corticosteroids or other traditional immunosuppressive agents and is associated with a range of side-effects (Braun et al. 2008; Korbet 2012). Recent studies indicate that cyclosporine and mycophenolate mofetil each induce remission in some steroid resistant patients (Hogg et al. 2013). Other novel agents including adalimumab (anti-TNF alpha), galactose and a novel dual endothelin receptor blocker and angiotensin receptor blocker (DUET trial, Sparsentan) are in trials (Joy et al. 2010; Trachtman et al. 2011a, b, 2015). A biomarker that could be used to stratify FSGS patients according to risk for rapid progression would be useful in design and interpretation of such trials. Such a biomarker might eventually allow individualization of therapy according to the potential risks and benefit.

Strengths of this study include the fact that our unique sample included FSGS patients who had reached RRT at the time of testing for P_alb_. All FSGS patients whose samples we analyzed were included; there were no censored observations. Patients represented a wide range of rates of progression and included both genders, and included both children and adults as well as several ethnicities. However, the study has several limitations. First, the dataset was limited to a cohort of FSGS patients who had progressed to RRT. We did not include samples from patients who continued to have adequate GFRs at the time the sample was submitted. We had only limited information regarding the patients’ prior history. Our information regarding medications was limited to their current therapy at the time of serum sample. We collected the information regarding current medications to examine potential interference with the P_alb_ activity assay. No other information about their treatment during the course of disease was available but it is likely that many patients had received corticosteroids. The low rates of immunosuppressive therapy may have reflected the fact that the majority had reached ESRD and were no longer on therapy directed at treatment of FSGS or nephrotic syndrome while others may never have received therapy. The small size of the sample does not permit analysis of potential medication effects but our prior work indicates that cyclosporine does not alter P_alb_ (Cattran et al. 2003). An additional limitation arises from the fact that the sample may reflect referral bias as it relates to submission of the sample for P_alb_ testing. The sample may include a disproportionate number of patients who had good access to health care and to physicians who were aware of the potential for P_alb_ testing. It may also over-represent patients with prior post-transplant recurrence or those who are awaiting initial renal transplants.

We have used P_alb_ to follow activity of plasma during sequential fractionation and to characterize the circulating permeability factor for recurrence of FSGS in allografts. The central role for a circulating permeability factor in FSGS is based on the observations of (a) early recurrence of nephrotic syndrome after transplantation, (b) development of albuminuria in rats after injection of serum from these patients, (c) response to plasma exchange and (d) occurrence of transient proteinuria in infants born to women with FSGS (Savin et al. 1996; Sharma et al. 2002, 2004a, b; McCarthy et al. 2010; Zimmerman 1984; Gohh et al. 2005; Kemper et al. 2001). We have described the circulating permeability factor as a hydrophobic sialoprotein with a molecular weight in the range of 30 kDa. It binds to galactose coated agarose beads and can be eluted using a galactose solution (Sharma et al. 2004a). We have used mass spectrometry to identify cardiotrophin-like cytokine factor-1 (CLCF-1) in active fractions and have shown that glomerular and podocyte responses to CLCF-1 parallel the responses to FSGS serum (McCarthy et al. 2010; Sharma et al. 2015). Another candidate for the FSGS “factor” has been identified by Wei and colleagues. They have reported that increased concentrations of soluble urokinase-type plasminogen activator receptor (suPAR) are present in FSGS plasma and propose that suPAR concentration predicts post-transplant recurrence (Wei et al. 2011). Additional molecules including hemopexin, angiopoietin-like-4 and CD80 are associated with nephrotic syndrome and have also been shown to cause proteinuria in experimental animals (Garin et al. 2010; Lennon et al. 2008; Clement et al. 2011) but do not appear to be relevant to FSGS. The severity of nephrotic syndrome or FSGS is generally classified as “steroid sensitive”, “steroid resistant” or “steroid dependent” and according to the response to cytotoxic or immunosuppressive agents. These categories are necessarily applied only post hoc and cannot be used to guide initial treatment. Likewise, even within categories defined by response to therapy, there is no clear association between clinical parameters and progression of renal failure. There are no reports of a quantitative method for stratifying risk of rapid progression to RRT.

We have proposed that P_alb_ activity ≥0.5 reflects the presence of a high concentration of circulating permeability factor(s) which causes significant glomerular injury and rapid progression in FSGS. P_alb_ activity was ≥0.5 in 38 % of 26 children with newly diagnosed nephrotic syndrome 2 of whom were later diagnosed with FSGS. In that sample, P_alb_ but did not differ between steroid sensitive and steroid resistant patients (Trachtman et al. 2004). We have found P_alb_ activity ≥0.5 in 20–38 % in additional cohorts of FSGS (Savin et al. 1996; Trachtman et al. 2004). We previously reported that patients in whom FSGS recurred after transplantation had a higher P_alb_ (0.47 ± 0.06) compared to that of FSGS patients who did not recur (0.14 ± 0.06). A P_alb_ value of 0.5 permitted risk stratification, with 86 % of patients with P_alb_ value of ≥0.5 developing recurrence versus only 17 % with P_alb_ < 0.5 (Savin et al. 1996). In an independent sample of patients awaiting transplantation, P_alb_ was greater than 0.6 in 9 of 10 patients with prior recurrence or rapid progression to ESRD (mean P_alb_ 0.76 ± 0.21, n = 10) (Gohh et al. 2005). Testing P_alb_ activity is currently available on a limited scale in our laboratory. It may provide an opportunity to evaluate FSGS patients using a non-invasive test. Measurement of a single molecule in the circulation may eventually supplant this functional assay, but the identity of active molecules in FSGS is currently being debated. In addition, any specific assay will risk missing a previously unidentified injurious molecule. The P_alb_ assay may be a valuable tool both in designing and interpreting clinical research trials on FSGS. Prospective validation of our findings in a trial with defined selection criteria will be required prior to the general use of P_alb_ activity for establishing prognosis in patients with FSGS.

 In summary, our results indicate that high P_alb_ activity is associated with rapid progression to RRT in biopsy proven FSGS. We suggest that P_alb_ activity may be useful for risk stratification in future clinical trials of aggressive therapy or novel therapy in FSGS. Further studies will be required before P_alb_ activity can be used to provide specific prognosis or dictate therapy in individual patients.
